# Microbiological and clinical effects of probiotics and antibiotics on nonsurgical treatment of chronic periodontitis: a randomized placebo- controlled trial with 9-month follow-up

**DOI:** 10.1590/1678-7757-2017-0075

**Published:** 2018-01-16

**Authors:** Alicia Morales, Alessandro Gandolfo, Joel Bravo, Paola Carvajal, Nora Silva, Claudia Godoy, Jocelyn Garcia-Sesnich, Anilei Hoare, Patricia Diaz, Jorge Gamonal

**Affiliations:** 1 Universidad de Chile, Facultad de Odontología, Departamento de Odontología Conservadora, Laboratorio de Biología Periodontal, Chile; 2 Universidad de Antofagasta, Facultad de Odontología, Departamento de Odontología, Chile; 3 Universidad de Chile, Facultad de Odontología, Departamento de Medicina y Patología Oral, Laboratorio de Microbiología, Chile; 4 Division of Periodontology, Department of Oral Health and Diagnostic Sciences, The University of Connecticut Health Center, Farmington, Connecticut, United States of America

**Keywords:** Chronic periodontitis, Lactobacillus rhamnosus, Azithromycin

## Abstract

**Objective:**

The aim of this double-blind, placebo-controlled and parallel- arm randomized clinical trial was to evaluate the effects of *Lactobacillus rhamnosus* SP1-containing probiotic sachet and azithromycin tablets as an adjunct to nonsurgical therapy in clinical parameters and in presence and levels of *Tannerella forsythia, Porphyromonas gingivalis* and *Aggregatibacter actinomycetemcomitans.*

**Material and Methods:**

Forty-seven systemically healthy volunteers with chronic periodontitis were recruited and monitored clinically and microbiologically at baseline for 3, 6 and 9 months after therapy. Subgingival plaque samples were collected from four periodontal sites with clinical attachment level ≥1 mm, probing pocket depth ≥4 mm and bleeding on probing, one site in each quadrant. Samples were cultivated and processed using the PCR technique. Patients received nonsurgical therapy including scaling and root planing (SRP) and were randomly assigned to a probiotic (n=16), antibiotic (n = 16) or placebo (n = 15) group. *L. rhamnosus* SP1 was taken once a day for 3 months. Azithromycin 500mg was taken once a day for 5 days.

**Results:**

All groups showed improvements in clinical and microbiological parameters at all time points evaluated. Probiotic and antibiotic groups showed greater reductions in cultivable microbiota compared with baseline. The placebo group showed greater reduction in number of subjects with *P. gingivalis* compared with baseline. However, there were no significant differences between groups.

**Conclusions:**

The adjunctive use of *L. rhamnosus* SP1 sachets and azithromycin during initial therapy resulted in similar clinical and microbiological improvements compared with the placebo group.

## Introduction

Chronic periodontitis is an inflammatory disease, produced in response to periodontopathogens in the biofilm of the subgingival plaque, affecting tissues supporting the teeth. The prevalence of this disease is close to 90% worldwide. In Chile, the destruction of periodontal tissues affects the majority of the adult population. The loss of clinical attachment higher than 3 mm in at least one site was of 93.4% for the population aging 35-44 years and of 97.5% for the group aging 65-74 years. When the severity of clinical attachment loss increased to 5 mm or more in at least one site, the percentage of the population affected was 58.3% and 81.4%, respectively^6^.

The etiology of this disease has been considered as polymicrobial, in which specific bacteria in the community have been associated with the development of the disease after the host defense response to the noxa. Evidence shows that the total bacterial load varies in healthy sites when compared with unhealthy sites^1^. Members of the red complex, such as *Porphyromonas gingivalis* and *Tannerella forsythia* described by Socransky, et al^23^(1998), are considered as the most pathogenic microbial components at present. The bacteria of the red complex present a similar prevalence in patients with different forms of periodontitis^23^. Likewise, *Aggregatibacter actinomycetemcomitans* is associated with periodontal disease, but it does not belong to the red complex.

In Chile, the prevalence of *P. gingivalis, A. actinomycetemcomitans* and *T. forsythia* in adults with chronic periodontitis was higher than 75%, 20% and 15% respectively^5^. In this context, periodontal therapy is focused on the control of the associated microbiota, removing or reducing the bacterial load of the periodontopathogens associated with the subgingival biofilm. The gold standard in periodontal treatment is formed by scaling and root planing^4^. Systemic antibiotic therapy is indicated to control deep periodontal pockets, difficult to access and with microbial invasion at epithelial level, with tissue destruction and disease progressing over time. The recolonization of other oral sites by periodontopathogens accounts for the failure of conventional therapy. Nevertheless, in Chile, there are no studies providing information about the response of the native microbiota to the systemic use of antibiotics. Therefore, it is important to consider the microbial resistance observed for the use of these antibacterial agents^17^.

Given the background in the literature, the selection of bacterial species resistant to the antibacterial treatment has been considered a global problem after the excessive use of these drugs. This leads to the search for new tools for the control of infectious diseases^8^. The use of probiotics has become more common in recent years. They are food supplements with microbial elements that have a physiologic effect on the organism that receives them. The effect of the use of probiotics in the treatment of chronic periodontitis had been studied^13^ ^,^ ^15^ ^,^ ^16^ ^,^ ^22^ ^,^ ^26^ ^,^ ^27^ ^,^ ^29^. *Lactobacillus* constitutes the most common bacterial genus used as a probiotic. *In vitro* studies have shown that oral strains of *Lactobacillus,* including *Lactobacillus rhamnosus*, display a strong inhibitory effect against the cariogenic species as well as against the Gram-negative periodontal pathogens^25^. Thus, the objective of our study was to evaluate the effects of *Lactobacillus rhamnosus* SP1 containing probiotic sachet and azithromycin tablets as an adjunct to nonsurgical therapy in clinical and microbiological parameters of chronic periodontitis.

## Material and methods

### Participant population and inclusion and exclusion clinical criteria

This study was carried out between June 2014 and August 2016. It is a double-blind, placebo-controlled and parallel-arm randomized clinical trial and it was conducted in accordance with the Helsinki Declaration of 1975, as revised in 2013. This clinical trial was approved by the local Research Ethics Committee of the Faculty of Dentistry, University of Chile (Decision no.: 2012/ 08). The protocol of the study was explained to all patients, who signed an informed consent form after explanation of the purpose, nature, risks and benefits of participating in this study (identification no. NCT02839408; http://www.clinicaltrials.gov).

Individuals in search of periodontal care or patients referred to the Diagnosis Center of the Faculty of Dentistry, University of Chile, for periodontal care were screened for the study. Ninety-six volunteers were initially examined, of which we included 47 in this study. Inclusion criteria were: healthy, non- institutionalized male or female subjects, at least 35 years of age, presence of a minimum of 14 natural teeth, excluding third molars, presence of at least 10 posterior teeth, previously untreated for periodontitis. Exclusion criteria were: suffering any systemic illness, pregnant and breastfeeding women, having received any periodontal treatment before the time of examination, having received antibiotics or non-steroid anti-inflammatory therapy in the 6-month period prior to the study. Chronic periodontitis was determined as follows: presence of at least five teeth with periodontal sites with pocket probing depth (PPD) ≥ 4mm and clinical attachment loss (CAL) ≥1 mm, 20% bleeding on probing (BOP) and extensive radiographically determined bone loss^28^.

### Experimental design: clinical trial

Sample size calculation, based on a study previously published^16^, was made for the primary outcome variable (CAL), considering a standard deviation of 1 mm and a difference between the groups of 1 mm. According to the calculation, 14 patients were needed in each group to provide 80% power with an a of 0.05.

After baseline examinations, all patients were given proper oral hygiene instructions, using standardized manual toothbrush. Scaling and root planing per quadrant was performed with one-week intervals in 4-6 sessions (by Paola Carvajal and Claudia Godoy). SRP was performed using an ultrasonic scaler (Cavitron, Dentsply, York, PA, U.S.A) and hand instruments (Hu Friedy Mfg. Co. Inc., Chicago, IL, U.S.A.). The study coordinator (Jorge Gamonal) randomized the participants over the three treatment groups: placebo (SRP + placebo), probiotic (SRP + probiotic) or antibiotic (SRP + antibiotic) group. According to gender, age, and smoking status, eligible individuals were randomly allocated to groups after the basal examination, using a computer-generated randomization table (Jorge Gamonal). Allocation concealment was prepared using sequentially numbered, opaque sealed envelopes. The probiotic group patients received *Lactobacillus rhamnosus* SP1 [(2×10^7^colony forming units (CFU)/ day)] (Macrofood S.A., Santiago de Chile, Chile) for 3 months. The dose was one sachet taken orally daily. The sachets presented to the patients were identical. Individuals were instructed to dissolve 1 sachet in water (150 mL) and ingest it once a day after brushing their teeth. Also, probiotic group patients received placebo with identical taste and appearance than antibiotic capsules. The antibiotic group patients took azithromycin 500 mg q.d, for 5 days and a probiotic placebo with identical taste, texture and appearance to the probiotic sachet. Placebo group patients received placebo from the manufacturer of identical taste, texture, and appearance to the probiotic sachet and antibiotic capsule. The patients started taking the probiotic, antibiotic or placebo after the last session of SRP. Every 3 months, they received periodontal supportive therapy (by Paola Carvajal and Claudia Godoy), with monitoring of individual compliance, medical history and diet throughout the study period. Patients, examiner and dentists who performed periodontal treatment were blinded to the study group assignment except for the study coordinator (Jorge Gamonal). The designation of the different groups was only revealed after study completion.

The study coordinator handed out the study materials.

### Clinical examination

Periodontal clinical examination consisted of full-mouth PPD, dichotomous measurements of supragingival plaque accumulation, and BOP at the base of the crevice, measured at six sites per tooth. CAL was determined using the distance from the cement-enamel junction (CEJ) to the free gingival margin (FGM) and the distance from the FGM to the bottom of the pocket/sulcus. All examinations were performed using a first generation manual periodontal probe (UNC-15, Hu Friedy Mfg. Co. Inc., Chicago, IL, U.S.A.) by one calibrated examiner (Alicia Morales) (intra-class correlation coefficient of 0.80 for CAL).

Clinical examination was recorded at baseline 3, 6 and 9 months after therapy.

### Subgingival plaque samples

Subgingival plaque samples were collected from four periodontal sites with clinical attachment level ≥ 1mm, probing pocket depth ≥ 4mm and bleeding on probing, one site in each quadrant. After isolating the area with cotton rolls and gently air-drying it, supragingival deposits were carefully removed with curettes (Hu Friedy Mfg. Co. Inc., Chicago, IL, U.S.A). Two standardized no. 30 sterile paper points (Johnson & Johnson, Tokyo, Japan) were inserted into the deepest part of the periodontal pocket for 20 seconds in order to obtain subgingival microbial samples. Each sample was deposited in a vial containing 1 ml of cold sterilized pre-reduced transport fluid (RTF) without EDTA. Vials with samples were transported at 4°C to the Microbiological Laboratory of the Faculty of Dentistry, University of Chile, and processed immediately.

Subgingival samples were collected at baseline 3, 6 and 9 months after therapy by one examiner (Jorge Gamonal).

### Microbiological procedures

Microbiological procedures were performed by one expert (Nora Silva).

Subgingival plaque samples were dispersed by mixing for 45 seconds followed by a 10-fold serial dilution of the bacterial suspension in RTF, using PBS.

Procedures to detect and quantify *P. gingivalis* and *T. forsythia* were: Aliquots of 100 μL of the appropriate dilution (10^-2^ and 10^-3^) were plated on nonselective blood- agar, hemin- menadione medium. Plates were anaerobically incubated at 35°C for 14 days in a jar containing gas generator envelopes for the production of an anaerobic atmosphere (Anaerogen. Oxoid Limited, Wade Road, Basingstoke, Hampshire, U.K.).

Procedures to detect and quantify A. *actinomycetemcomitans* were: Aliquots of 100 μL of the appropriate dilution (undiluted and 10^−1^) were plated on selective TSBV medium (trypticase, 10% horse serum, bacitracin, and vancomycin). Plates were incubated at 37°C for 2 to 3 days in CO_2_ candle jars.

Bacteria were primarily identified by colony morphology under a stereoscopic microscope (Stmi 2000-C, Zeiss, Jena, Germany), pigment production and Gram stain. In addition, black pigmented colonies were tested for red fluorescence under UV light (360 nm) and methanol-negative result indicated that colonies were *Porphyromonas* spp. *A. actinomycetemcomitans* was also primarily identified by colony morphology and catalase production.

Using direct method, total cultivable microbiota (total microbial load) was count on blood-agar, hemin-menadione medium and TSBV medium. The percentage of *P. gingivalis* and *T. forsythia* was obtained using the number of CFU/ml RTF on blood- agar hemin- menadione medium as a percentage of the total anaerobic count. The percentage of *A. actinomycetemcomitans* was obtained using the number of CFU/ml RTF on TSBV as a percentage of the total anaerobic counts.

Final identification was made using PCR according to Ashimoto protocol.

### Outcome variables

The primary outcome variable was change in CAL. Secondary outcome variables were changes in PPD, PI and BOP, total cultivable microbiota, percentage of *P. gingivalis, T. forsythia* and *A. actinomycetemcomitans,* and prevalence of *P. gingivalis, T. forsythia* and *A. actinomycetemcomitans.*

### Compliance and adverse reactions

The participant returned the sachets containing probiotic or placebo at 1, 2 and 3-month visit. Each time, patients received new sachets. Antibiotic group participants returned azithromycin tablets at 6-week visit. All participants were called by phone each week to check for compliance. In each control visit or phone call, the clinical examiner (Alicia Morales) inquired the participants regarding general health changes, use of mouth rinses, use of probiotic products and any adverse events.

### Statistical analysis

For all statistical assessments, the patient was maintained as the unit of measurement. The compliance of parameters to the normal distribution was assessed using Shapiro Wilk test. The balancing of groups by age, sex and smoking was tested by Kruskall Wallis, ANOVA and Fisher's exact tests. We recorded quantitative data as the mean value ± standard deviation or median, measured the IQ score by using the Friedman test, and we used McNemar test to compare intragroup parameters. For both tests, the statistical significance was set at p<0.05. We used the Bonferroni- corrected Wilcoxon signed ranks test to evaluate intragroup comparisons. Bonferroni- corrected Kruskal Wallis, ANOVA and Fisher's exact tests were used to compare intergroup parameters. The statistical significance was set at p<0.017 for all the Bonferroni- corrected tests.

The statistical analysis was made using a statistical package (StataCorp, College Station, TX, U.S.A)

## Results

**Figure 1:**
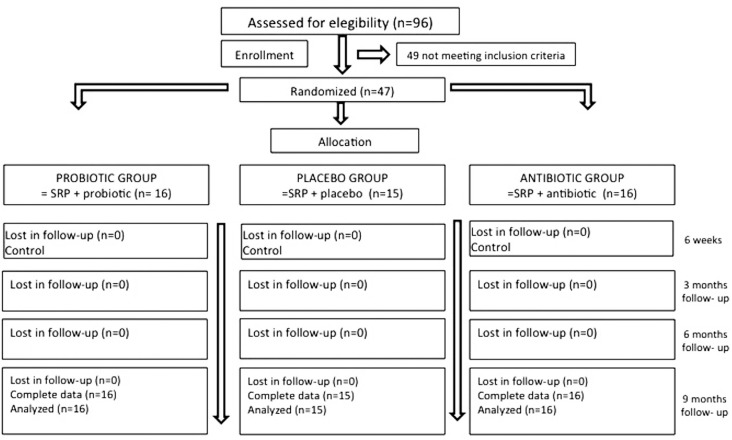
Flowchart of the study design

The flow chart of the study is shown in Figure 1. Forty-seven patients, 16 in the probiotic group, 16 in the antibiotic group and 15 in the placebo group were analyzed. All patients entering the study also completed it. No compliance problems were noted, all patients followed the protocol of the study. Only one subject from the antibiotic group reported an adverse event (nausea).

### Intergroup analysis

At baseline, no significant differences in demographic, medical and clinical characteristics were found between groups (p>0.05, Table 1). Also, there were no intergroup differences in CAL, PPD, BOP, plaque accumulation, total cultivable microbiota and percentages of *P. gingivalis, A. actinomycetemcomitans* and *T. forsythia* at 3, 6, and 9 months follow-up (Table 2 and 3).

**Table 1:** Baseline data of patients in the treatment groups

Variable	Treatment Group			p- value
	Probiotic group (n=16)	Antibiotic group (n=16)	Placebo group (n=15)	
Age^1^ (years)	46.5 ± 9.3	49.0 ± 7.9	52.8 ± 7.5	0.1171
Gender (M/F)^2^	8 / 8	10 / 6	8 / 7	0.8150
Smokers^2^	7 (43.8%)	3 (18.7%)	6 (40.0%)	0.3440
CAL (mm)^3^	3.8 ± 0.7	4.4 ± 0.9	4.7 ± 1.5	0.0824
PPD (mm)^3^	2.7 ± 0.6	2.9 ± 0.4	3.1 ± 0.9	0.2437
BOP (%)^1^	49.3 ± 18.1	57.4 ± 10.2	52.5 ± 12.6	0.0850
Plaque accumulation (%)^3^	54.5 ± 18.8	58.6 ± 18.8	56.1 ± 9.4	0.5256

1 ANOVA (p<0.05);

2 Fisher's exact test (p<0.05);

3 Kruskal Wallis test (p<0.05).

CAL: Clinical attachment level; PPD: Probing pocket depth; BOP: Bleeding on probing.

**Table 2:** Intra- and intergroup comparisons of clinical and microbiological parameters (mean ± SD or median, IQ score)

		Probiotic group					Antibiotic group					Placebo group			
		(n=16)					(n=16)					(n=15)			
	Baseline	3 months	6 months	9 months	P	Baseline	3 months	6 months	9 months	P	Baseline	3 months	6 months	9 months	P
CAL (mm)	3.8 ± 0.7	3.4±0.6†	3.3 ±0.6	3.4±0.6†	0.0001*	4.4 ±0.9	4.0 ± 1.0†	3.9 ± 1.0†	4.1 ±1.0	0.0001*	4.7 ± 1.5	4.1 ± 1.4†	4.1 ± 1.4†	4.3 ± 1,4†	0.0001*
PPD (mm)	2.7 ± 0.6	2.1 ±0.3†	2.3 ± 0.4†	2.2±0.3†	0.0001*	2.9 ±0.4	2.3 ± 0.4†	2.3 ± 0.3†	2.3 ± 0.3†	0.0004*	3.1 ± 0.9	2.4±0.5†	2.4±0.5†	2.5 ± 0.6†	0.0001*
BOP(%)	49.3 ± 18.1	39.2 ± 14.8	42.1 ± 13.6	42.4 ± 14.6	0.0010*	57.4 ± 10.2	43.6 ± 12.5†	44.1 ± 13.5†	48.1 ± 14.1	0.0128*	52.5 ± 12.6	40.7 ± 13.3†	41.5 ± 14.5†	45.9 ± 12.9	0.0002*
Plaque accumulation (%)	54.5 ± 18.8	24.7 ± 11.3†	25.2 ± 13.1†	28.1 ± 14.6†	0.0023*	58.6 ± 18.8	28.0 ± 17.5†	32.6 ± 15.7†	31.8 ± 14.8†	0.0001*	56.1 ± 9.4	32.4 ± 13.9†	28.7 ± 13.0†	27.4 ± 13.0†	0.0119*
Total cultivable microbiota (CFU)* 105	7.6, 31.8	0.5, 1.3	0.8, 1.7†	0.6, 2.9	0,0619	10.2, 23.5	1.1,3.0†	1.9, 4.0†	2.2, 5.9†	0,0937	10.0, 20.2	2.9, 25.4	0.5, 8.1	2.8, 24.7	0,1445

Intra-group comparison by

* Friedman test (p<0.05) and intra- group multiple comparison by

† Bonferroni- corrected Wilcoxon signed rank test (p<0.017). Significant values were given in bold formatting.

Inter-group multiple comparison by

** Bonferroni- corrected Kruskal Wallis test and ANOVA (p<0.017)

CAL: Clinical attachment level; PPD: Probing pocket depth; BOP: Bleeding on probing; CFU: Colony forming units

**Table 3:** Intra- and intergroup comparisons of microbiological parameters (mean ± SD or median, IQ score at days 0 and 270

Variable	Time point			Treatment group				p- value**	
		Probiotic group (n=16)		Antibiotic group (n=16)		Placebo group (n=15)		For mean	For delta
		Mean ± SD	Delta ± SD	Mean ± SD	Delta ± SD	Mean ± SD	Delta ± SD		
% *P. gingivalis*									
	Day 0	14.2 ± 17.6	-13.1 ± 18.6	17.8 ± 19.3	-15.6 ± 17.9	14.7 ± 15.7	-14.4 ± 15.9	0.97738	0.99028
	Day 270	1.1 ± 2.6†		2.1 ± 6.6†		0.3 ± 1.0†		0.5882	
Number of subjects with									
*P. gingivalis*									
	Day 0	15 (93.7%)		14 (87.5%)		13 (86.6%)		0.8590	
	Day 270	9 (56.2%)		9 (56.3%)		3 (20.0%)†		0.0740	
*A. actinomycetemcomitans*									
	Day 0	3 (18.7%)		3 (18.7%)		0 (0%)		0.2200	
	Day 270	1 (6.3%)		6 (37.5%)		5 (33.3%)		0.0860	
*T. forsythia*									
	Day 0	16 (100%)		16 (100%)		15 (100%)		1.0000	
	Day 270	12 (75.0%)		11 (68.7%)		10 (66.6%)		0.9240	

Intra- group comparison by

* Wilcoxon signed rank test and Mc Nemar test. p<0.05. Significant values were given in bold formatting. Inter-group comparison by

** Kruskal Wallis test, ANOVA and Fisher's exact test. p<0.05

### Intragroup analysis

The comparison of CAL, PPD, BOP, plaque accumulation values for the baseline and 3-, 6-, and 9-month time points for all groups are presented in Table 2. In the probiotic group we observed a significant reduction of the clinical attachment loss at 3 and 9 months and of the PPD and plaque accumulation at all times of the follow-up. In the antibiotic group, we perceived a significant reduction of the CAL and BOP at 3 and 6 months, as well as a significant reduction of the PPD and plaque accumulation at all times of the follow-up. Finally, in the placebo group, the CAL, PPD and plaque accumulation decreased significantly at all times and the BOP at 3 and 6 months.

Also, we observed a variation of the total cultivable microbiota, as seen in Table 2. Compared with baseline, there was a significant reduction in the probiotic group at 6-month follow-up, while for the antibiotic group it occurred at all times (p<0.017).

In Table 3, we analyzed the variation of the microbiological variables between the basal time and the 9-month follow-up. Percentage of *T. forsythia* and *A. actinomycetemcomitans* is not reported, because the development of these microorganisms was not observed at any time of the analysis. The percentage of *P. gingivalis* (p<0.05) decreased in all the groups, compared with baseline. The reduction of the number of subjects with *P. gingivalis* was significant only in the placebo group (p<0.05).

## Discussion

This double-blind, placebo-controlled and parallel- arm randomized clinical trial evaluated clinical and microbiological effects of *L. rhamnosus* SP1 administered one time a day for 3 months and azithromycin, in addition to nonsurgical therapy in chronic periodontitis. Our results showed that the adjunctive use of *L. rhamnosus* sachets and azithromycin during initial therapy resulted in similar periodontal clinical improvements compared with mechanical therapy alone. At microbiological level, the total cultivable microbiota decreased significantly in the probiotic and antibiotic groups. In the placebo group, the prevalence of subjects with *P. gingivalis* decreased. However, there were no significant differences between groups.

To our knowledge, this is the first study assessing and comparing the microbiological impact of the use of probiotics and antibiotics on the treatment of chronic periodontitis with a 9-month follow-up. In the probiotic group, we observed attachment gain, reduction of PPD, and reduction of plaque, which was not significant when compared with the other groups. This is partially consistent with studies using as probiotics: *Lactobacillus reuteri* DSM-17938 + ATCC PTA 5289^27^, *Streptococcus oralis* KJ3 + *Streptococcu uberis* KJ2 + *Streptococcu rattus* JH145^13^ and *L. rhamnosus* SP1^16^, but contrary to studies that also used *L. reuteri* strains^26^ ^,^ ^29^, as well as *Lactobacillus salivarius* WB21^22^, in which the probiotic group presented additional beneficial effects when compared with the placebo group.

Regarding the use of azithromycin in the treatment of chronic periodontitis in our study, a significant reduction in all periodontal parameters was observed, without intergroup differences, though. This is consistent with the studies by Sampaio, et al.^20^ (2011), Han, et al.^10^ (2012) and Hincapie, et al.^12^ (2014), who concluded that there are no additional effects of azithromycin to the nonsurgical periodontal therapy. However, some studies show that the antibiotic group presents a significant gain of attachment together with a significant reduction of BOP and PPD, when compared with the placebo group^7^ ^,^ ^14^ ^,^ ^18^ ^,^ ^30^.

Regarding microbiological parameters, the total cultivable microbiota decreased in the probiotic group at 6-month follow-up, but without significant intergroup differences. This is consistent with the studies that used *L. reuteri, Streptococcus* and *L. salivarius.* Tekce, et al.^26^ (2015) reported an insignificant reduction of the percentage of obligate anaerobes at 1-year follow-up in the probiotic group *(L. reuteri* DSM17938 and ATCCPTA5289) and placebo. The administration of a probiotic with *Streptococcus* caused a significant reduction of CFU/ml of *T. forsythia, P. gingivalis, A. actinomycetemcomitans, Fusobacterium nucleatum* and *Prevotella intermedia* in the probiotic and placebo groups at 3-month follow-up, but without significant differences between groups^13^. The intake for 8 weeks of *L. salivarius* WB 21 did not generate significant differences in the quantification of *A. actinomycetemcomitans, P. intermedia, P. gingivalis, T. denticola,* and *T. forsythia* in patients with chronic periodontitis, smokers and nonsmokers^15^. In other studies, the evidence is contrary. Furthermore, in the study by Teughels, et al.^27^ (2013), samples of subgingival plaque were taken at 3, 6, 9 and 12 weeks of intake of probiotic *L. reuteri* DSM17938 and ATCC PTA5289, identifying and quantifying in real time by PCR *T. forsythia, P. gingivalis, A. actinomycetemcomitans, Fusobacterium nucleatum* and *Prevotella intermedia.* After 12 weeks of treatment with probiotic, a significant reduction of the quantification of the periodontopathogens selected in both groups was reported. However, in the probiotic group, the variation in the colony numbers was significantly higher than in the placebo group after 9 weeks of treatment. In the study by Vivekananda, et al.^29^ (2010), the quadrants of patients who took probiotics *(L. reuteri* DSM-17938 + ATCC PTA 528), regardless whether they were treated or not, presented a reduction in the number of UFC/ml of *P. gingivalis, A. actinomycetemcomitans* and *P. intermedia,* at the end of the 3-week intake of the probiotic.

In our study, we observed a reduction of the total cultivable microbiota at all follow-up times in the antibiotic group, but there were no significant differences with the other groups. Gomi, et al.^7^ (2007) reported no intergroup differences in the prevalence of *P. gingivalis, T. forsythia* and *A. actinomycetemcomitans* between the antibiotic and the placebo groups. Yashima, et al.^30^ (2009) reported no significant differences between the study groups regarding the total count of bacteria at 12-month follow-up and the prevalence of *P. gingivalis, T. forsythia* and *A. actinomycetemcomitans.* Sampaio, et al.^20^ (2011) reported no significant differences in the prevalence and count between the experimental and control groups in any periodontopathogen at any time of the study. In another trial carried out by Han, et al.^10^ (2012), both groups had a similar percentage of periodontopathogens at all the times. There were no significant intergroup differences for *P. gingivalis, T. forsythia* and *A. actinomycetemcomitans.* On the other hand, Sefton, et al.^21^ (1996) reported a significant reduction in the total count of microorganisms, black- pigmented bacteria and *P. gingivalis* in the antibiotic group versus the placebo group. Haffajee, et al.^8^ (2008) found a significant difference in the count of red-complex bacteria and some species of the orange complex for the group treated with azithromycin. Oteo, et al.^18^ (2010) studied the impact of the treatment with the mentioned antibiotic in patients with chronic periodontitis with presence of *P. gingivalis.* In this experimental group, a significant reduction in the frequency of detection of *P. gingivalis* and *A. actinomycetemcomitans* at 6-month follow-up and of *T. forsythia* at 1-month follow-up were reported.

However, the identification and quantification of the presence of periodontopathogens are not enough, since according to the genetic variability of their virulence factors, they present resistance to relatively high concentrations (200 μg/mL) of Polymyxin B. It is a synthetic cationic peptide, used as the gold standard of the antimicrobial activity of the endogenous cationic peptide, such as the human β defensins^2^, a situation that could be replicated in the case of the azithromycin. The microorganism can also present a different immunogenicity^3^.

The selection of the "best" probiotic for oral health is still a controversial topic. In addition to this, evidence coming from gastroenterology has started a change in the concept of probiotics. The treatment with antibiotic destroys the bacterial populations forming the commensal microbiota, which generates a dysbiosis, increasing the susceptibility to a wide range of other bacterial infections, as it reduces the resistance to colonization. Specifically, the destruction of obligate anaerobes coming from the lower gastrointestinal tract results in the expansion of oxygen-tolerant bacteria such as γ-proteobacteria and *Enterococcus spp.* This bacterium has been identified in oral diseases, including chronic periodontitis, and presents, among others, virulence factors associated with resistance to antibacterial treatments, becoming a reservoir of transferable elements that would favor the genetic variability associated with microbial resistance^24^. Hence, it has been proposed that the development of commensal bacteria as probiotics is a high priority for preventive and therapeutic purposes^19^.

The use of antibiotic therapy as an adjunct to the treatment of periodontal disease is widely supported in the literature, and there is evidence that it provides additional beneficial effects to mechanical therapy^9^. However, the optimal usage protocol for antibiotic therapy and the clinical effects of the time at which antibiotic is administered during the course of the periodontal hygienic phase has not yet been clearly determined^11^. According to the literature, administration of antibiotics show higher clinical results when is accompanied by meticulous disruption and mechanical removal of the periodontal biofilm^11^. In our study, the patients started taking the probiotic, antibiotic or placebo after the last session of SRP. We selected *L. rhamnosus* as the probiotic for our study because it has been shown to have good antimicrobial activity against the Gram-negative periodontal pathogen^25^. Mode of administration, dosage and frequency may also affect therapy outcomes. In our study the *L. rhamnosus* sachet application [(2×10^7^ colony forming units (CFU/day)] was started immediately after the last session of root planing, one time a day for 3 months. Teughels, et al^27^. (2013) used *L. reuteri* lozenges two times a day for 3 months, 1×10^8^ CFU/day, immediately after a full-mouth disinfection procedure.

The major limitation of our study is the statistical power. This study could be too small to detect the real differences between the groups. An increase of the sample size is suggested.

In conclusion, the administration of *L. rhamnosus* SP1 in sachets and azithromycin in pills for the treatment of chronic periodontitis generates clinical and microbiological effects similar to the SRP on its own.
